# Impact of Inducible Nitric Oxide Synthase Activation on Endothelial Behavior under Magnesium Deficiency

**DOI:** 10.3390/nu16101406

**Published:** 2024-05-07

**Authors:** Giorgia Fedele, Sara Castiglioni, Valentina Trapani, Isabella Zafferri, Marco Bartolini, Silvana M. Casati, Pierangela Ciuffreda, Federica I. Wolf, Jeanette A. Maier

**Affiliations:** 1 Department of Biomedical and Clinical Sciences, Università di Milano, 20157 Milano, Italy; giorgia.fedele@unimi.it (G.F.); sara.castiglioni@unimi.it (S.C.); isabella.zafferri@unimi.it (I.Z.); marco.bartolini@unimi.it (M.B.); silvana.casati@unimi.it (S.M.C.); pierangela.ciuffreda@unimi.it (P.C.); 2 Alleanza Contro il Cancro, Viale Regina Elena 299, 00161 Rome, Italy; trapani@alleanzacontroilcancro.it; 3 Department of Medicine, Saint Camillus International Medical School (UniCamillus), Via di Sant’Alessandro 8, 00131 Rome, Italy; federica.wolf@unicamillus.org

**Keywords:** magnesium, magnesium deficiency, ROS, HUVEC, nitric oxide

## Abstract

Endothelial dysfunction is a crucial event in the early pathogenesis of cardiovascular diseases and is linked to magnesium (Mg) deficiency. Indeed, in endothelial cells, low Mg levels promote the acquisition of a pro-inflammatory and pro-atherogenic phenotype. This paper investigates the mechanisms by which Mg deficiency promotes oxidative stress and affects endothelial behavior in human umbilical vascular endothelial cells (HUVECs). Our data show that low Mg levels trigger oxidative stress initially by increasing NAPDH oxidase activity and then by upregulating the pro-oxidant thioredoxin-interacting protein TXNIP. The overproduction of reactive oxygen species (ROS) activates NF-κB, leading to its increased binding to the inducible nitric oxide synthase (iNOS) promoter, with the consequent increase in iNOS expression. The increased levels of nitric oxide (NO) generated by upregulated iNOS contribute to disrupting endothelial cell function by inhibiting growth and increasing permeability. In conclusion, we provide evidence that multiple mechanisms contribute to generate a pro-oxidant state under low-Mg conditions, ultimately affecting endothelial physiology. These data add support to the notion that adequate Mg levels play a significant role in preserving cardiovascular health and may suggest new approaches to prevent or manage cardiovascular diseases.

## 1. Introduction

Magnesium (Mg), an essential mineral critical for various physiological functions in humans, significantly impacts cardiovascular health by regulating vascular tone, endothelial function, and blood pressure [1]. Accordingly, Mg deficiency is implicated in endothelial dysfunction, oxidative stress, inflammation, and impaired vasodilation [2], key early events in the pathogenesis of cardiovascular diseases, including hypertension, atherosclerosis, and coronary artery disease. Endothelial cells, which line the inner walls of blood vessels, are highly responsive to changes in Mg levels. There exists substantial evidence indicating a profound relationship between Mg levels and endothelial cell function. Despite the growing recognition of Mg’s significance in preserving endothelial health, the precise molecular mechanisms linking Mg deficiency to endothelial dysfunction remain incompletely understood. The acute impact of Mg was explored in human umbilical vascular endothelial cells (HUVECs) under low-Mg conditions (0.1 mM) for 24 h, utilizing whole genome transcription analysis [3]. A total of 2728 transcripts (5.8% of the transcripts) exhibited differential expression in low Mg vs. the physiological (1.0 mM) control cultures. This study reaffirms Mg’s critical role in inflammation [4] and underscores its substantial contribution to essential physiological pathways, such as intracellular signaling, metabolic activities, and survival pathways [3], which further strengthens the ever-growing scientific evidence that Mg deficiency adversely affects the function of cultured HUVECs [2]. Indeed, low Mg levels promote oxidative stress [5] and the activation of NF-κB, which fosters the acquisition of a pro-inflammatory and pro-atherogenic phenotype [2]. Oxidative stress is in part attributed to the upregulation of the thioredoxin-interacting protein (TXNIP), which inhibits the antioxidant function of thioredoxin (TRX), a protein responsible for scavenging reactive oxygen species (ROS); this results in the accumulation of ROS and cellular stress [6]. Elevated TXNIP levels are associated with the onset of endothelial senescence [7] and hypertension-induced endothelial dysfunction [8]. Despite its significant role in ROS generation within the vasculature, data regarding the activity of pro-oxidant enzyme NADPH oxidases in HUVECs exposed to Mg-deficient medium are currently unavailable. Regarding the connection between low Mg and inflammation in HUVECs, NF-κB activation by low Mg leads to increased monocytes adhesion to HUVECs by upregulating the vascular cell adhesion molecule (VCAM) [2], inducing the plasminogen activator inhibitor (PAI) 1 and promoting the synthesis of pro-inflammatory cytokines, among which is interleukin (IL)-1 alpha [2], which results in retarded proliferation and triggers senescence [2]. Mg deficiency also alters endothelial barrier function, and as such, it may contribute to the early pathogenesis of atherosclerosis [6]. Notably, endothelial function is significantly impaired in a model of inherited hypomagnesemia in mice (MgL mice) [9].

Mg influences the activity of nitric oxide (NO) synthase and the synthesis/release of NO, a small, highly reactive signaling molecule intricately involved in a variety of physiological processes and a key modulator of vascular homeostasis. NO contributes to maintaining vascular tone and possesses anti-platelet and anti-thrombotic properties [10]. Endothelial cells express a constitutive form of NO synthase (endothelial NO synthase, eNOS), which regulates NO generation under physiological conditions. However, upon inflammatory stimulation, NO accumulates because of the activation of inducible NOS (iNOS). Controlled production of NO favors a coordinated regulation of coagulation, inflammation, and vascular tone, but excessive NO levels can have adverse results, including a reversible increase in endothelial permeability [11]. It is known that high extracellular Mg levels stimulate NO production by enhancing eNOS levels [2], while no data are available about the effects of low Mg on NO synthesis in HUVECs.

In this study, our aims were (i) to unravel the mechanisms involved in fostering oxidative stress in HUVECs cultured in Mg-deficient medium and (ii) to explore whether alterations in NO synthesis occur and influence endothelial behavior.

## 2. Materials and Methods

### 2.1. Cell Culture

HUVECs were from the American Type Culture Collection (ATCC, Manassas, WV, USA) and cultured on 2% gelatin-coated dishes (Euroclone, Milan, Italy), as described in [6]. These cells are broadly utilized as an experimental model of macrovascular endothelial cells to study cardiovascular and metabolic diseases [12].

A Mg-free medium was purchased from Invitrogen (Thermo Fisher Scientific, Waltham, MA, USA) and supplemented with MgSO_4_ to obtain the final concentrations of 0.1 mM or 1.0 mM Mg. All the other culture reagents were from Gibco-Invitrogen (Thermo Fisher Scientific, Waltham, MA, USA). In all the experiments, the cells were seeded in normal growth medium and grown for 24 h before switching the medium to either 0.1 mM or 1.0 mM Mg-containing medium. The physiological concentration of Mg is ~1.0 mM. In humans, the lowest Mg level observed in critically ill patients is ~0.4–0.5 mM [13]. In rodents, in a widely used experimental model to study the cardiovascular effects of Mg deficiency, an 8-day experimental diet led to a reduction in plasma Mg levels to 0.14 mM [14]. Based on these observations and on the reports in literature, we employed Mg concentrations of 0.1 mM to simulate Mg deficiency in vitro and used the physiological 1.0 mM as the control.

In some experiments, HUVECs were pre-treated with apocynin (10 μM) or N-acetyl-l-cysteine (NAC, 1 mM) (Sigma Aldrich, St. Louis, MO, USA).

Transient downregulation of iNOS was achieved by using stealth siRNAs (20 nmol, 5′-ATCGAATTTGTCAACCAATAT-3′, Invitrogen) and Lipofectamine RNAiMAX (Invitrogen, Thermo Fisher Scientific, Waltham, MA, USA) [2], as recommended by the manufacturer. After 6 h, the siRNA transfection medium was replaced with control or Mg-deficient medium for an additional 24 h. Scrambled non-silencing (NS) sequences were used as controls. To assess cell proliferation, the cells were seeded at low density (7500/cm^2^) and silenced as described above. After 24 h or 72 h, viable cells were identified by trypan blue staining (0.4%) and counted using an automatic cell counter (Logos Biosystems, Annandale, VA, USA). Endothelial permeability was assessed using the Transwell Permeability Assay (Transwell Permeable Supports, 0.4 µm micropores, Euroclone, Pero, MI, Italy) as described [2]. Briefly, confluent HUVECs seeded into the inserts were maintained in 0.1 mM Mg + NS, 1 mM Mg + NS, or 0.1 mM Mg + siRNA iNOS for 24 h. Leakage of fluorescein isothiocyanate-labeled albumin (FITC–BSA) (1 mg/mL, Sigma Aldrich, St. Louis, MO, USA) through the monolayer from the upper to the lower comportment was determined by measuring the fluorescence using a Varioskan LUX Multimode Microplate Reader (Thermo Fisher Scientific, Waltham, MA, USA) (λexc = 495 nm, λem = 519 nm).

All the experiments were performed in triplicate and repeated at least three times with similar results. Data are expressed as mean ± standard deviation (SD).

### 2.2. ROS Measurement

HUVECs were cultured in a black 96-well plate (Greiner bio-one, Frickenhausen, Germany) and incubated for 30 min with 2′-7′-dichlorofluorescein diacetate (DCFDA, 10 mM) solution (Thermo Fisher Scientific, Waltham, MA, USA) to detect total intracellular ROS. ROS levels were determined from fluorescence emission, as detailed in [5]. The results were expressed as the mean ± SD of three independent experiments, each conducted in triplicate.

### 2.3. Determination of NADPH Oxidase Activity

HUVECs were cultured in either 0.1 or 1.0 mM Mg-containing medium for 90 min. NADPH oxidase activity was measured using a lucigenin-derived chemiluminescence assay (Promega, Madison, WI, USA). Briefly, total protein cell homogenates were diluted in Krebs buffer composed of 140 mmol/L NaCl, 5 mmol/ L KCl, 2.5 mmol/L CaCl_2_, 1 mmol/L Na_2_HPO_4_, 1 mmol/L MgSO_4_, 5.5 mmol/L glucose, and 0.026 mmol/L EDTA (pH 7.4) and distributed (50 μg/well) on the microplate. NADPH (100 μmol/L, Sigma Aldrich, St. Louis, MO, USA) and lucigenin (5 μmol/L, Sigma Aldrich, St. Louis, MO, USA) were added into the well just before reading. Luminescence was measured every minute for 5 min with the Varioskan LUX Multimode Microplate Reader (Thermo Fisher Scientific, Waltham, MA, USA) in order to measure relative changes in NADPH oxidase activity.

### 2.4. Reduced to Oxidized Glutathione (GSH/GSSG) Ratio

Cells were cultured in either 0.1 or 1.0 mM Mg-containing medium for 4 and 24 h. The luminescence-based GSH/GSSG-Glo Assay (Promega, Madison, WI, USA) was used to measure reduced glutathione (GSH) and oxidized glutathione (GSSG) according to the manufacturer’s instructions. Data are shown as percentages of GSH/GSSG levels in HUVECs cultured in 0.1 vs. 1.0 mM Mg-containing media. The results were expressed as the mean ± SD of three independent experiments, each conducted in triplicate.

### 2.5. Determination of NO Levels

After deproteination, NOS activity was measured in the media by using the Griess method, as described [11], and by using gas chromatography mass spectrometry (GC-MS) [15]. The calculation of concentrations of nitrites and nitrates was accomplished as described [15] and normalized on the cell number. The results were expressed as the mean ± SD of three independent experiments, each conducted in triplicate.

### 2.6. Real-Time PCR

RNA was extracted as detailed in [6]. Single-stranded cDNA was synthesized from 0.2 μg, and real-time PCR was performed using the CFX96 Real-Time PCR System instrument (Biorad, Hercules, CA, USA) using TaqMan Gene Expression Assays (Life Technologies, Thermo Fisher Scientific, Waltham, MA, USA): iNOS (Hs01075529_m1); the internal reference gene was GAPDH (Hs99999905_m1). Relative changes in gene expression were analyzed by the 2^−ΔΔCt^ method [6]. The experiments were repeated three times and expressed as the mean ± SD.

### 2.7. Western Blot Analysis

Cell extracts (80 µg/lane) were resolved on 8% SDS-PAGE, transferred to nitrocellulose sheets at 250 mA for 16 h, and probed with the antibodies anti-iNOS (BD Biosciences, Milano, Italy), eNOS/p-Ser^1177^ (Cell Signaling Technology, Danvers, MA, USA), eNOS (BD Biosciences), TXNIP, and actin (Santa Cruz Biotechnology, Dallas, TX, USA). The SuperSignal chemiluminescence kit (Thermo Fisher Scientific, Waltham, MA, USA) was used to detect immunoreactive proteins. All the Western blots were repeated at least three times, and densitometry was performed by the ImageJ software (Java 1.8.0_241).

### 2.8. NF-κB Activation by TransAM Assay

HUVECs were pre-treated with apocynin and NAC for 60 min and then cultured in either 0.1 or 1.0 mM Mg-containing medium for an additional 90 min. The TransAM assay was performed according to the manufacturer’s instructions (Active Motif, Carlsbad, CA, USA). Briefly, 5 μg of nuclear extract was added into the wells of a 96-well plate, where binding between nuclear NF-κB (if present in the extract) and the NF-κB consensus site (immobilized on the well bottom) could occur. Binding was then detected by indirect immunofluorescence, with a primary anti-NF-κB p65 antibody and a secondary horseradish-peroxidase-conjugated antibody. The colorimetric readout, representing specific binding, was measured by a spectrophotometer (450 nm) and expressed as arbitrary units. The results are represented as the mean ± SD of three separate experiments in triplicate.

### 2.9. Statistical Analysis

Results are shown as means ± SD. The data were normally distributed and were analyzed using one-way repeated measures ANOVA. The *p*-values deriving from multiple pairwise comparisons were corrected by the Bonferroni method. Statistical significance was defined for *p*-values ≤ 0.05, and * *p* ≤ 0.05; ** *p* ≤ 0.01; and *** *p* ≤ 0.001.

## 3. Results

### 3.1. Mg Deficiency Increases ROS Production by Activating NADPH Oxidase and Upregulating TXNIP

**Figure 1:**
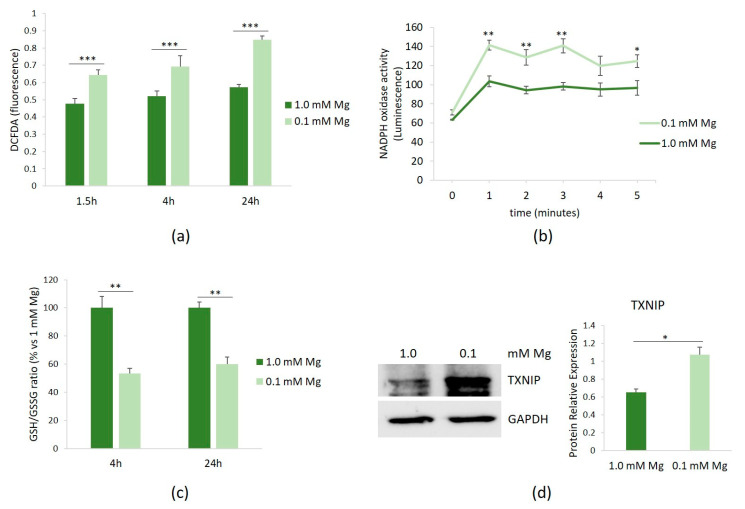
ROS in HUVECs cultured in low Mg. HUVECs were cultured in 0.1 or 1.0 mM Mg-containing medium. All the experiments were performed in triplicate at least three times. (**a**) After 1.5, 4, and 24 h of culture in media containing 0.1 or 1.0 mM Mg, ROS were measured as described in the methods. (**b**) After 1.5 h of culture in media containing 0.1 or 1.0 mM Mg, NADPH oxidase activity was measured using a lucigenin-derived chemiluminescence assay. (**c**) The GSH/GSSH ratio was measured using a luminescence-based assay. The data were expressed as the percentage vs. 1.0 mM Mg ± SD. (**d**) The total amounts of TXNIP were evaluated by Western blot after 20 h of exposure to low-Mg medium. Anti-GAPDH antibodies were used as a control of equal loading. A representative blot and densitometry performed on three independent experiments and obtained by ImageJ are shown. * *p* ≤ 0.05; ** *p* ≤ 0.01; and *** *p* ≤ 0.001.

HUVECs were cultured in 0.1 or 1.0 mM Mg-containing medium for 1.5, 4, and 24 h. Then, the amounts of ROS were measured using the DCFDA probe. Figure 1a shows that Mg-deficient medium led to a rapid accumulation of ROS, which persisted for up to 24 h.

We then attempted to identify the intracellular sources of ROS in HUVECs cultured in low-Mg medium. Since NADPH oxidase serves as an important source of ROS in vascular cells, we assessed its activity in HUVECs cultured in either 0.1 or 1.0 mM Mg-containing medium. After 1.5 h of culture in Mg-deficient medium, NADPH oxidase activity was increased, as demonstrated using a lucigenin-derived chemiluminescence assay (Figure 1b). In addition, because GSH is the predominant antioxidant in aerobic cells [16], we measured the GSH/GSSG ratio and observed that it significantly decreased after 4 and 24 h of culture in low-Mg medium (Figure 1c). We also confirmed the upregulation of the pro-oxidant TXNIP after 20 h of exposure to low-Mg medium (Figure 1d).

### 3.2. Antioxidants Prevent the Activation of NF-κB

**Figure 2:**
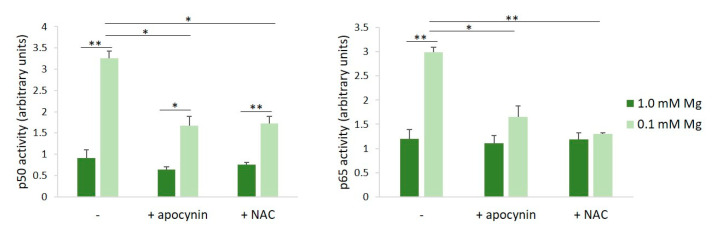
Inhibition of low-Mg-induced NF-κB activation by apocynin and NAC in HUVECs cultured in low-Mg conditions. The cells were pre-incubated with apocynin or NAC for 60 min. Then, the medium was discarded, and fresh medium containing either 0.1 or 1.0 mM Mg added with apocynin (10 µM) or NAC (1 mM) was added for an additional 90 min. p50 and p65 activities were quantified by TransAM NF-κB analysis on nuclear extracts. Results are expressed as arbitrary units and represent the mean ± SD of three separate experiments in triplicate. * *p* ≤ 0.05; ** *p* ≤ 0.01.

ROS serve as common intracellular messengers of NF-κB activation, which triggers the transcription of genes involved in the inflammatory response, among which is iNOS [17]. We asked whether the inhibition of ROS production prevented NF-κB activation in HUVECs cultured in Mg-deficient medium. We used apocynin, which exhibits intrinsic antioxidant properties, acting as a scavenger for ROS, and NAC, required for the synthesis of GSH [18]. HUVECs were pre-treated with apocynin or NAC for 60 min before exposure to either 0.1 or 1.0 mM Mg-containing medium for 90 min. We determined NF-κB activation by assessing the activities of p65 and p50 in HUVECs cultured in 0.1 or 1.0 mM Mg using the TransAM assay. Figure 2 shows that the incubation of HUVECs in low-Mg conditions increases p65 and p50 nuclear activity, but this effect is reverted by antioxidants.

### 3.3. Mg Deficiency Modulates the Release of NO through the Upregulation of iNOS

**Figure 3:**
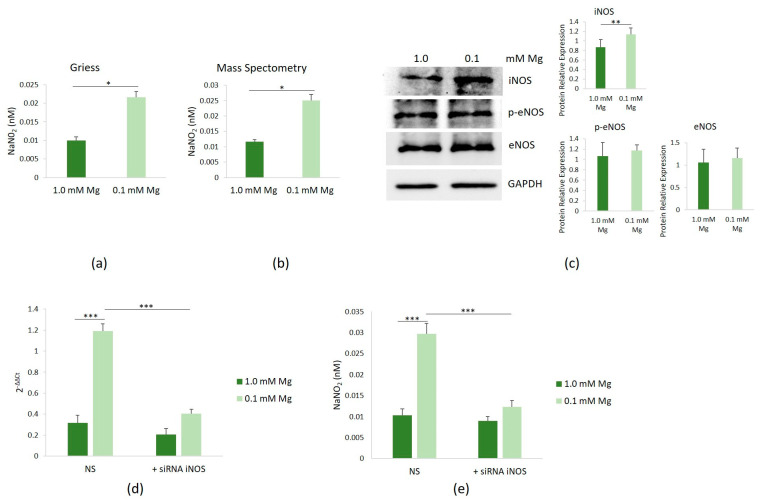
Induction of iNOS and NO synthesis in HUVECs cultured in low-Mg conditions. HUVECs were cultured in 0.1 or 1.0 mM Mg-containing medium for 24 h. All the experiments were performed in triplicate at least three times. NO levels in culture medium were measured by the Griess method (**a**) and mass spectrometry (**b**). (**c**) Western blot was performed to evaluate the total amounts of iNOS, eNOS, and eNOS/p-Ser^1177^ (p-eNOS). Anti-GAPDH antibodies were used as a control of equal loading. A representative blot and densitometry performed on three independent experiments and obtained by ImageJ are shown. (**d**) The mRNA expressions of iNOS were analyzed by real-time PCR. (**e**) NO levels were measured in the medium of the cells using the Griess method after silencing iNOS. Non-silencing scrambled sequences (NS) were used as controls. * *p* ≤ 0.05; ** *p* ≤ 0.01; and *** *p* ≤ 0.001.

We then investigated whether low Mg affected NOS activity in HUVECs cultured in Mg-deficient medium. Following 24 h of culture in media containing either 0.1 or 1.0 mM Mg, NOS activity was found to be elevated in HUVECs exposed to 0.1 mM Mg, compared to controls, as evidenced by the Griess assay (Figure 3a) and confirmed through mass spectrometry analysis (Figure 3b). Notably, both methods yielded consistent results. Therefore, we used the Griess method in all the following studies.

To elucidate which isoform of NOS contributed to the increased NO levels observed upon culture in Mg-deficient medium, we assessed the total amounts of iNOS and eNOS, the two enzymes responsible for NO production in endothelial cells. Since the phosphorylation of eNOS-Ser^1177^ has been shown to enhance enzyme activity [19], we also determined the levels of eNOS/p-Ser^1177^. Using Western blot, we consistently observed a significant upregulation of iNOS in HUVECs cultured in 0.1 mM Mg-containing medium for 24 h, while the levels of eNOS and eNOS/p-Ser^1177^ remained unchanged (Figure 3c). To uncover the mechanism underlying iNOS upregulation, we conducted RT-PCR and detected a substantial increase in the iNOS transcript in cells cultured in 0.1 mM Mg-containing medium (Figure 3d). iNOS silencing reduced the levels of the transcript, with respect to cells transfected with non-silencing scrambled sequences used as controls, and reduced NO levels as well (Figure 3e).

### 3.4. The Role of NO in Mediating Endothelial Response to Low Mg

**Figure 4:**
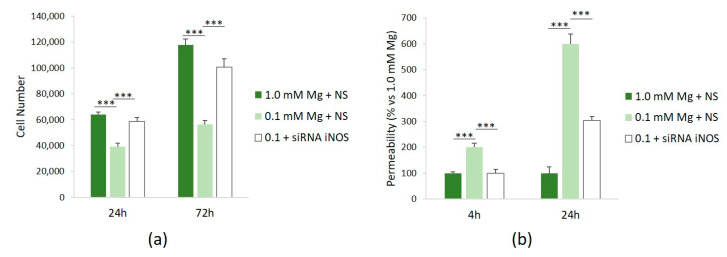
NO mediates low-Mg effects on cell proliferation and permeability. All the experiments were performed in triplicate at least three times. HUVECs were cultured in 1.0 mM Mg + NS, 0.1 mM Mg + NS, or 0.1 mM Mg + siRNA iNOS. (**a**) Cells were counted after 24 and 72 h using an automatic cell counter. (**b**) Permeability was measured using the Transwell method after 24 h. The data are expressed as the percentage vs. *** *p* ≤ 0.001.

To assess the role of NO in mediating some of the effects of Mg deficiency in HUVECs, we investigated cell proliferation and permeability after silencing iNOS. Figure 4a shows that siRNA against iNOS rescues the low-Mg-induced retardation of cell proliferation after 24 and 72 h. We also demonstrated that silencing iNOS prevents hyperpermeability in cells cultured in low Mg (Figure 4b).

## 4. Discussion

Mg has long been recognized as an essential macronutrient involved in vital processes; however, the impact of its deficiency on health is underestimated. Because of decreased Mg content in crops, the loss of Mg during food processing and refining, and medications, about two-thirds of individuals in developed nations do not meet their minimum daily dietary Mg requirement [20]. Reduced Mg intake has been associated with chronic inflammation and heightened oxidative stress [4]. Multiple surveys have shown that dietary Mg is inversely correlated with plasma levels of the C-reactive protein, an inflammation marker, and thiobarbituric acid reactive substances, indicative of lipid peroxide content [21]. At the interface between blood and tissue, endothelial cells rapidly sense Mg fluctuations and inflammatory mediators. Although many studies report the onset of endothelial dysfunction under Mg restriction [2], the mechanisms involved are not completely unveiled. Here, we demonstrated the central role of NO generated by the upregulation of iNOS in mediating the effects of low Mg in human endothelial cells. NO, a short-lived radical, serves as a signaling molecule governing various functions, from the regulation of vascular tone to neurotransmission, immune response, and oxidation-sensitive mechanisms. Vascular endothelial cells continuously produce NO, which relaxes smooth muscle cells, prevents platelet activation, and controls endothelial cell permeability and adhesiveness [10]. Constitutively synthesized NO, through eNOS, maintains the barrier function and does not impact cell proliferation. On the contrary, the activation of iNOS and the subsequent overproduction of NO inhibit cell growth without affecting cell viability [22]. Of note, hypercholesterolemia, mechanical injuries, and inflammatory mediators, all involved in atherogenesis, upregulate iNOS [23]. Accordingly, iNOS is overexpressed in human coronary atherosclerotic plaques [24]. Low Mg is an additional factor triggering the upregulation of iNOS in endothelial cells, in part through transcriptional regulation. Indeed, we demonstrated that the excessive NO resulting from iNOS upregulation is the cause of barrier dysfunction and growth retardation, important aspects in atherogenesis, in HUVECs cultured in low-Mg conditions. Of interest, Mg deficiency also enhances NO production via iNOS in alveolar macrophages [25] and in osteoblasts, where it is involved in braking cell growth [26]. An intriguing issue is that when cultured in high-Mg conditions, HUVECs also produce more NO, primarily due to elevated eNOS activity [2]. Under these conditions, the cells exhibit enhanced proliferation. In brief, through different mechanisms, both low and high Mg increase NO synthesis, but the impact on endothelial behavior is diametrically opposed. It is only under low-Mg conditions that pathways leading to the accumulation of ROS are activated. It can therefore be hypothesized that ROS and NO synergize to generate a highly pro-oxidative environment, which ultimately slows down HUVECs growth. It is feasible that high NO levels in HUVECs might generate peroxynitrite (ONOO^−^) by reacting with superoxide, further aggravating the redox imbalance induced by low Mg. While it is well accepted that Mg deficiency is associated with oxidative stress in vivo and in vitro, the mechanisms involved have not been completely clarified. Here, we provided evidence that multiple mechanisms contribute to generate a pro-oxidant state under low-Mg conditions. We previously excluded the mitochondria as a source of ROS in HUVECs cultured in low Mg [6], which is not surprising, considering that about 80% of ATP comes from glycolysis rather than from the electron transport chain [27]. Here, we showed the activation of NADPH oxidase, the principal oxidase system responsible for oxidative stress in vascular diseases, in HUVECs cultured in Mg-deficient medium [28]. NADPH oxidase activation leads to the formation of superoxide and hydrogen peroxide (H_2_O_2_), which then activate other enzymatic systems, leading to the secondary overproduction of ROS [28]. The activation of NADPH oxidase also results in eNOS uncoupling, an event that further increases ROS production [28]. Moreover, in Mg-deficient HUVECs, we confirmed the upregulation of TXNIP, which promotes oxidative stress by inhibiting thioredoxin scavenging activity [29] and modulates inflammatory responses [30]. Also, the GSH/GSSG ratio, a commonly used sensitive biomarker of the redox status [31], is reduced in cells cultured in low Mg, as previously reported in red blood cells from Mg-deficient rats [32].

**Figure 5:**
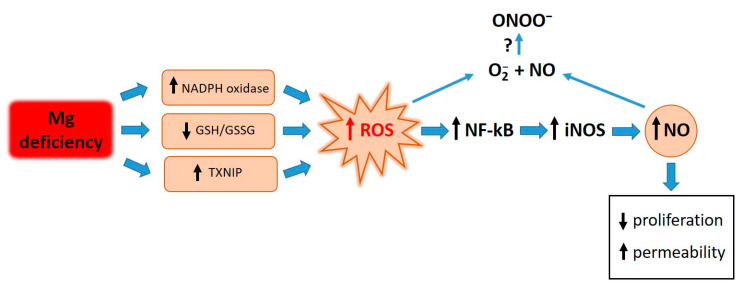
Graphical summary. Low-Mg conditions promote a highly pro-oxidant environment through various pathways. High ROS levels activate NF-kB, which in turn fuels inflammatory responses, including the upregulation of iNOS. ROS and NO contribute to retarding cell proliferation and inducing hyperpermeability. Further experiments are necessary to measure ONOO^-^ under our experimental conditions.

Elevated concentrations of ROS are responsible for the activation of NF-κB. Similarly to what occurs after treating endothelial cells with LPS and TNF-alpha [33], antioxidants prevent the activation of NF-κB in HUVECs cultured in low-Mg conditions. We therefore propose that the accumulation of ROS in Mg-deficient HUVECs activates NF-κB, which in turn fuels inflammatory responses, including the upregulation of iNOS (Figure 5).

## 5. Conclusions

The role of Mg deficiency in promoting cardiovascular diseases is supported by clinical studies [34]. By elucidating the intricate relationship between Mg deficiency and endothelial cell function, this paper seeks to provide insights into novel approaches for preventing or managing cardiovascular diseases by optimizing Mg status through an appropriate and balanced diet, rich in Mg, with the aim of preserving endothelial health.

## Data Availability

The raw data supporting the conclusions of this article will be made available by the authors on request.
